# Characterization of the In Vivo and In Vitro Metabolites of Linarin in Rat Biosamples and Intestinal Flora Using Ultra-High Performance Liquid Chromatography Coupled with Quadrupole Time-of-Flight Tandem Mass Spectrometry

**DOI:** 10.3390/molecules23092140

**Published:** 2018-08-25

**Authors:** Xinchi Feng, Yang Li, Chenxi Guang, Miao Qiao, Tong Wang, Liwei Chai, Feng Qiu

**Affiliations:** 1 School of Chinese Materia Medica, Tianjin University of Traditional Chinese Medicine, Tianjin 300193, China; xiaochi0211@163.com (X.F.); yangli940206@163.com (Y.L.); guangcx1994@163.com (C.G.); qmiao0319@163.com (M.Q.); wangtong_tj@sina.com (T.W.); chailiwei_tjtcm@126.com (L.C.); 2 Tianjin State Key Laboratory of Modern Chinese Medicine, Tianjin University of Traditional Chinese Medicine, Tianjin 300193, China

**Keywords:** linarin, ring cleavage metabolites, UPLC/Q-TOF-MS/MS, rats, intestinal flora

## Abstract

Linarin, a flavone glycoside, is considered to be a promising natural product due to its diverse pharmacological activities, including analgesic, antipyretic, anti-inflammatory and hepatoprotective activities. In this research, the metabolites of linarin in rat intestinal flora and biosamples were characterized using ultra-high-performance liquid chromatography/quadrupole time-of-flight mass spectrometry (UPLC/Q-TOF-MS/MS). Three ring cleavage metabolites (4-hydroxybenzoic acid, 4-hydroxy benzaldehyde and phloroglucinol) were detected after linarin was incubated with rat intestinal flora. A total of 17 metabolites, including one ring cleavage metabolite (phloroglucinol), were identified in rat biosamples after oral administration of linarin. These results indicate that linarin was able to undergo ring fission metabolism in intestinal flora and that hydrolysis, demethylation, glucuronidation, sulfation, glycosylation, methylation and ring cleavage were the major metabolic pathways. This study provides scientific support for the understanding of the metabolism of linarin and contributes to the further development of linarin as a drug candidate.

## 1. Introduction

Linarin (acacetin-7-*O*-β-d-rutinoside) is a naturally occurring flavonoid and it has been isolated from several medicinal plants, such as *Flos chrysanthemiindici*, *Buddleja officinalis*, *Cirsium setosum*, *Mentha arvensis* and *Buddleja davidii*. Various pharmacological studies have demonstrated linarin’s diverse pharmacological activities, for example its analgesic, antipyretic, anti-inflammatory and hepatoprotective activities, acetylcholinesterase and aldose reductase inhibitory activities, and sedative, neuroprotective and anti-apoptotic effects [1,2,3,4,5,6,7]. Our previous pharmacokinetic study showed that the absolute bioavailability of linarin was 0.47%, which indicated that linarin underwent extensive metabolism after oral administration [8]. Thus, a comprehensive metabolism study of linarin is needed and the detailed metabolic fate of linarin will provide the scientific basis for the development of linarin as a potential therapeutic.

To the best of our knowledge, to date only two studies associated with the metabolism of linarin have been completed. The identification and structural elucidation of the metabolites of linarin in rat urine was reported in our previous study using liquid chromatography-ion trap mass spectrometry (LC-IT-MS^n^) [9]. Six urinary metabolites were identified, urine being the only matrix evaluated. Information about the metabolites in other biosamples, such as plasma, feces and bile is still unavailable. In another study, in vitro biotransformation of linarin by the bacteria isolated from human feces was investigated and another five metabolites were reported [10]. However, all the metabolites identified in the above-mentioned studies possessed the intact flavone skeleton of two phenolic rings connected by a three-carbon unit. Thus far, no ring cleavage metabolites (rcMs) of linarin have been reported. 

As already frequently reported, many flavonoids can be transformed by the intestinal flora into a wide range of low-molecular-weight phenolic acids [11,12]. It is a general belief that these ring cleavage metabolites can be absorbed and several of the bioactivities of flavonoids are due to these metabolites [13,14]. In order to fully understand the metabolic fate of linarin, the following questions should be addressed: (1) Whether linarin can be metabolized into low-molecular-weight phenolic acids by intestinal flora; and (2) whether these ring cleavage metabolites can be detected in vitro. Thus, in this study, linarin was first incubated with rat intestinal flora and a high resolution ultra-performance liquid chromatography coupled with quadrupole time-of-flight mass spectrometry (UPLC/Q-TOF-MS/MS) was employed to identify the ring cleavage metabolites of linarin. Additionally, the metabolites of linarin in rat biosamples, including plasma, urine, bile and feces, were characterized in order to elaborate the comprehensive metabolic profile of linarin in vitro.

## 2. Results and Discussion

**Figure 1:**
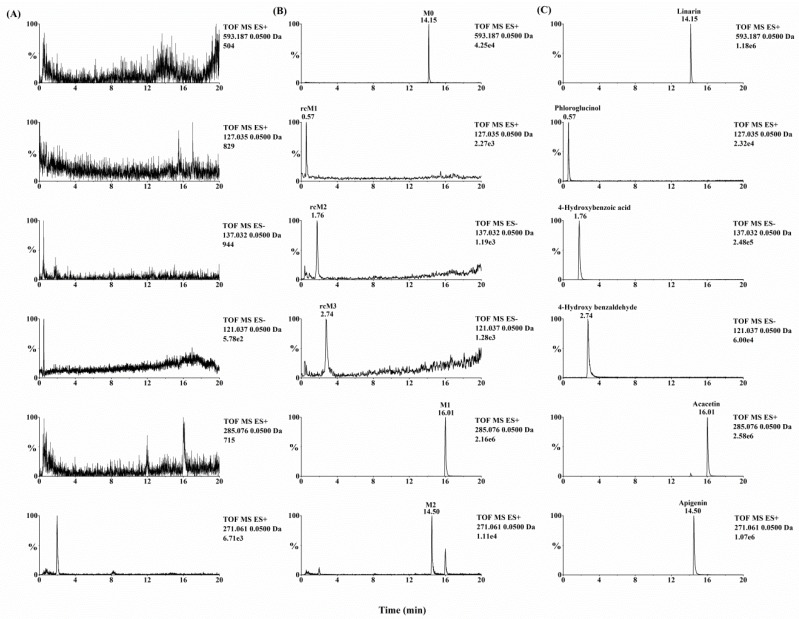
Extracted ion chromatograms of (**A**) linarin metabolites in rat intestinal flora incubated without linarin, (**B**) linarin metabolites in rat intestinal flora incubated with linarin and (**C**) reference standards. Compound abbreviations: M0, linarin; M1, acacetin; M2, apigenin; rcM1, phloroglucinol; rcM2, 4-hydroxybenzoic acid; rcM3, 4-hydroxy benzaldehyde.

**Figure 2:**
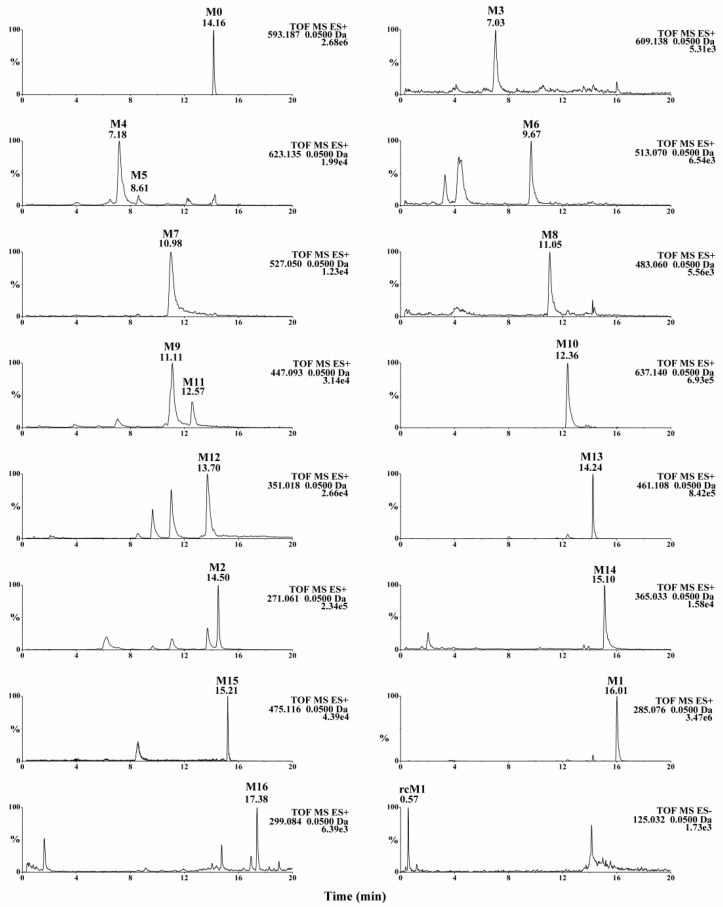
Extracted ion chromatograms (EICs) of linarin metabolites in rat biosamples. EICs for M0 and M16 were obtained from feces samples. EICs for M1-M15 were obtained from urine samples. EIC for rcM1 was obtained from bile sample. The compound abbreviations are explained in Table 1. EICs of linarin metabolites in blank rat biosamples were showed in Figure S1.

Metabolynx XS software (version 4.1) was applied and the formulae for the structures of of linarin, acacetin (the aglycone of linarin) and apigenin (a demethylation product of acacetin) were input into the software to elucidate the metabolism of linarin. By comparing the accurate molecular masses and mass fragmentations of the metabolites with those of linarin and reference standards, five metabolites, including three ring cleavage metabolites (Figure 1, Table 1), were identified after linarin was incubated with intestinal flora, and a total of 17 metabolites of linarin, including one ring cleavage metabolite (Figure 2, Figure S1, Table 1), were identified in rat urine, feces, plasma and bile.

### 2.1. Mass Spectral Analysis of Linarin and Acacetin

**Figure 3:**
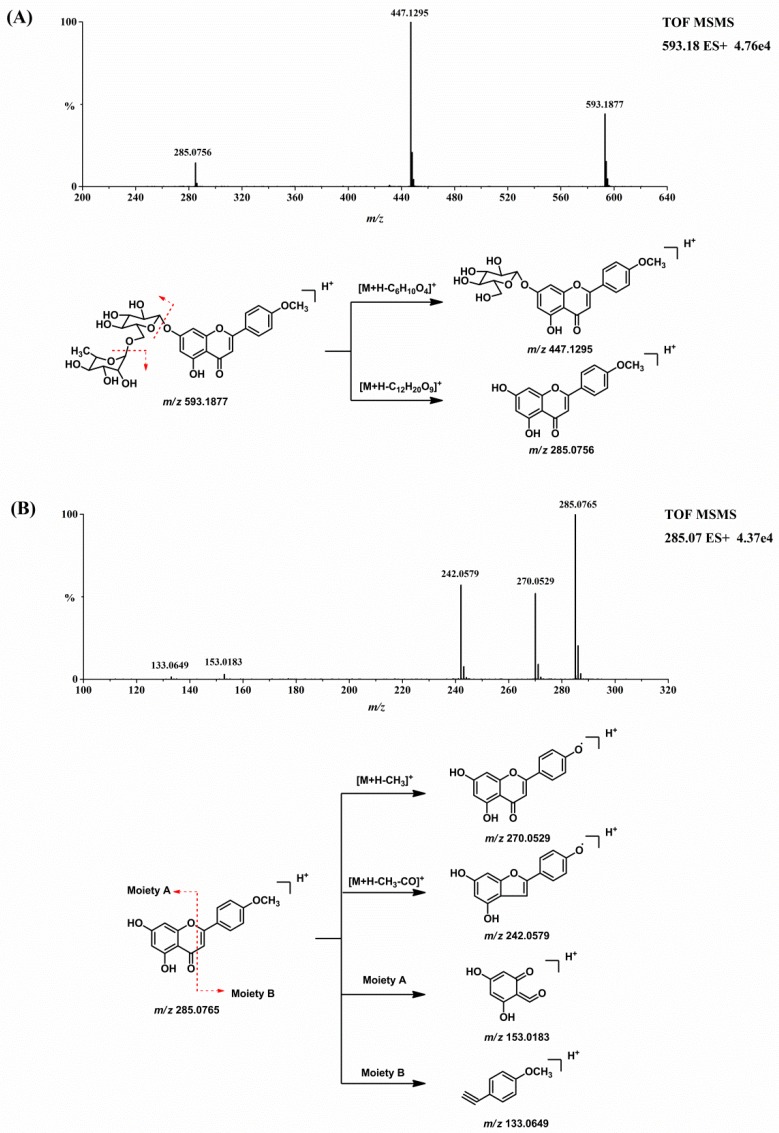
MS/MS product ion spectrum and the proposed fragmentation pathways of linarin (**A**) and acacetin (**B**).

Before the metabolites of linarin were characterized, the chromatographic and mass spectrometric fragmentation behaviors of linarin and acacetin (the aglycone of linarin) were investigated. The retention time of linarin (M0) was 14.16 min under the employed chromatographic conditions. In positive ion mode, the protonated molecular ion of linarin at *m/z* 593.1877 gave rise to product ions at *m/z* 447.1295 and 285.0756 by the loss of sugar moieties. Acacetin was eluted at 16.01 min with an accurate protonated molecular ion of *m/z* 285.0765, suggesting an elemental composition of C_16_H_12_O_5_. The [M + H]^+^ ion of acacetin generated product ions at *m/z* 270.0529 [M + H − CH_3_]^+^, 242.0579 [M + H − CH_3_ − CO]^+^, 153.0183, and 133.0649. The product ions at *m/z* 153.0183 and 133.0649 were attributed to the cross-ring cleavage [15]. Figure 3 shows the MS/MS product ion spectrum and the proposed fragmentation pathways of linarin and acacetin. These characteristic product ions and neutral losses from the parent compound can be used to identify the metabolites of linarin.

### 2.2. Metabolites of Linarin in Rat Intestinal Flora

Quadrupole time-of-flight mass spectrometry (QTOF-MS/MS) was run in both positive and negative ion modes to acquire valuable information about linarin’s metabolites. After linarin was incubated with rat intestinal flora, five metabolites were detected. Two of them (M1 and M2) showed better intensity in positive ion mode and another three metabolites (rcM1, rcM2 and rcM3) showed better intensity in negative ion mode.

#### 2.2.1. Acacetin (M1) and Apigenin (M2)

Acacetin (M1) and apigenin (M2) were two metabolites with an intact flavone skeleton. The retention time of M1 was 16.01 min and it exhibited the protonated ion at *m/z* 285.0771 (C_16_H_12_O_5_). Characteristic ions at *m/z* 270.0529, 242.0579, 153.0183 and 133.0649 suggested that M1 was acacetin, the aglycone of linarin. M1 was identified as acacetin by comparing the retention time and accurate mass spectrum with that of the authentic standard. M2, with the retention time of 14.50 min, exhibited the protonated molecular ion at *m/z* 271.0606, which was 14 Da less than that of acacetin. The characteristic ions at *m/z* 242.0572 and 153.0180 indicated that M2 possess a similar structure to acacetin. The diagnostic ion at *m/z* 119.0491 was 14 Da less than that of the product ion of acacetin at *m/z* 133.0649, suggesting that M2 was the demethylation product of acacetin (apigenin). This result was compared to the apigenin standard and M2 was identified as apigenin with regard to retention time and MS fragmentations.

#### 2.2.2. Ring Cleavage Metabolites

Three ring cleavage metabolites (rcM1, rcM2 and rcM3) were detected after linarin was incubated with intestinal flora. In negative ion mode, the retention time of rcM1 was 0.57 min and rcM1 exhibited a deprotonated ion at *m/z* 125.0245 (C_6_H_6_O_3_). RcM2, with a retention time of 1.76 min, exhibited a deprotonated ion at *m/z* 137.0237 (C_7_H_6_O_3_). For rcM2, a fragment ion at *m/z* 93.0333 (C_6_H_6_O) was generated via the loss of CO_2_. RcM3 showed a deprotonated ion at *m/z* 121.0285 (C_7_H_6_O_2_). Unfortunately, no stable MS/MS fragments were found for rcM1 and rcM3 and this might be due to the fact that the molecular weights of rcM1 and rcM3 were too low to generate stable product ions under our experimental conditions. According to the literature, flavone can undergo ring fission metabolism and its metabolites are phloroglucinol and hydroxyphenylpropionic acid derivatives [16]. Thus, rcM1, rcM2 and rcM3 were identified as phloroglucinol, 4-hydroxybenzoic acid and 4-hydroxy benzaldehyde by comparing their results with the relevant reference standards.

### 2.3. Metabolites of Linarin in Rat Biosamples

A total of 17 metabolites were detected in rat biosamples, including acacetin (M1), apigenin (M2) and phloroglucinol (rcM1), suggesting that the intestinal flora metabolites should be able to be detected in vivo. The identification of other metabolites was elucidated as follows.

#### 2.3.1. Methylation Metabolites

M16 exhibited a protonated ion at *m/z* 299.0924 (C_17_H_14_O_5_) and it was 14 Da higher than that of acacetin. The retention time of M16 was 17.38 min, which indicated that M16 eluted later than acacetin. The characteristic ion at *m/z* 284.2952 was yielded via the neutral loss of CH_3_ (15 Da). Thus, M16 was identified as the methylation product of acacetin.

#### 2.3.2. Glucuronidation Metabolites

M3 eluted at 7.03 min and showed a protonated molecular ion at *m/z* 609.1445 (C_27_H_28_O_16_). The fragment ion at *m/z* 447.0918 was yielded via the neutral loss of 162 Da (C_6_H_10_O_5_, 609.1445−447.0918 = 162), indicating that the bound sugar in M3 was glucose. The fragment ion at *m/z* 271.0604 was yielded via the neutral loss of one glucuronic acid moiety from the fragment ion at *m/z* 447.0918. Therefore, M3 was identified as the glucuronide conjugate of apigenin-7-glucoside.

M13 showed a protonated molecular ion at *m/z* 461.1083 (C_22_H_20_O_11_), which was 176 Da higher than that of acacetin. Characteristic neutral loss of 176 Da (*m/z* 285.0765 = 461.1083 − 176) in the MS/MS spectrum indicated that M13 was the glucuronidated metabolite of acacetin. M10 exhibited a protonated ion at *m/z* 637.1399 (C_28_H_28_O_17_), which was 352 Da (2 × 176 Da, 637.1399 − 285.0768 = 352) higher than that of acacetin. In the MS/MS spectrum, a fragment ion at *m/z* 461.1090 was yielded via the neutral losses of one glucuronic acid moiety and a fragment ion at *m/z* 285.0768 was generated via the neutral losses of two glucuronic acid moieties. Thus, M10 was determined to be the bis-glucuronide conjugate of acacetin.

M9 showed the protonated molecule at *m/z* 447.0923 (C_21_H_18_O_11_), which was 176 Da (447.0923 − 271.0605 = 176) higher than that of apigenin. M11 showed the same protonated molecule at *m/z* 447.0919. In their MS/MS spectra, fragment ions were yielded at *m/z* 271.0605 via the neutral losses of one glucuronic acid moiety. M9 and M11 were identified as the glucuronidated metabolites of apigenin. Similarly, M4 and M5 were identified as bis-glucuronide conjugates of apigenin and M15 was the glucuronidated product of M16.

#### 2.3.3. Sulfation Metabolites

M6 eluted at 9.67 min and showed a protonated molecular ion at *m/z* 513.0697 (C_21_H_20_O_13_S). The fragment ion at *m/z* 351.0166 was yielded via the neutral loss of 162 Da (C_6_H_10_O_5_, 513.0703 − 351.0166 = 162), indicating that the bound sugar in M6 was glucose. The fragment ion at *m/z* 271.0606 was yielded via the neutral loss of SO_3_ (80 Da). Therefore, M6 was identified as the sulfate conjugate of apigenin-7-glucoside.

M14 showed a protonated molecular ion at *m/z* 365.0317 (C_16_H_12_O_8_S), which was 80 Da higher than that of acacetin. A characteristic ion at *m/z* 285.0777 was yielded via the neutral loss of SO_3_ (80 Da, 365.0317 − 285.0777 = 80). The neutral loss of SO_3,_ together with other characteristic ions at *m/z* 270.0531, 242.0652 and 133.0989, indicated that M14 was the sulfate conjugate of acacetin. Similarly, M12 was identified as the sulfate conjugation product of apigenin.

M7 was eluted at the retention time of 10.98 min and it exhibited the protonated ion at *m/z* 527.0495 (C_21_H_18_O_14_S). A characteristic ion at *m/z* 447.0938 was yielded via the neutral loss of SO_3_ (80 Da, 527.0495 − 447.0938 = 80). A fragment ion was generated at *m/z* 351.0175 via the neutral loss of a glucuronic acid moiety (176 Da, 527.0495 − 351.0175 = 176). Another characteristic ion was yielded at *m/z* 271.0606 via the neutral loss of SO_3_ and a glucuronic acid moiety. Thus, M7 was identified as sulfate and glucuronide conjugation product of apigenin. 

#### 2.3.4. Glycosylation Metabolites

M8 showed a protonated ion at *m/z* 483.0601 (C_20_H_18_O_12_S). Diagnostic ions at *m/z* 271.0606 and 351.0171 indicated that M8 was the metabolite of the sulfate conjugation of apigenin. A characteristic ion was yielded at *m/z* 351.0171 via the neutral loss of 132 Da (C_5_H_8_O_4_, 483.0601 − 351.0171 = 132), which indicated that M8 was the glycosylation product by adduct formation with xylose. Thus, M8 was identified as the sulfate conjugation of the glycosylated apigenin.

### 2.4. Metabolic Pathways of Linarin

**Figure 4:**
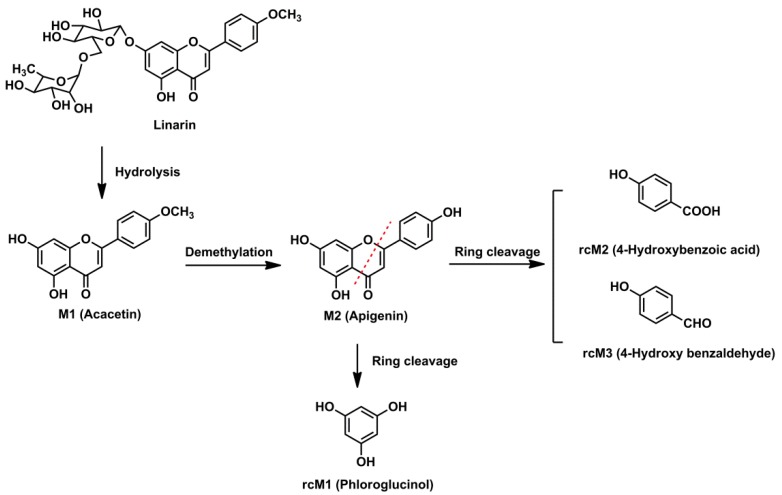
Proposed metabolic pathways of linarin in rat intestinal flora.

**Figure 5:**
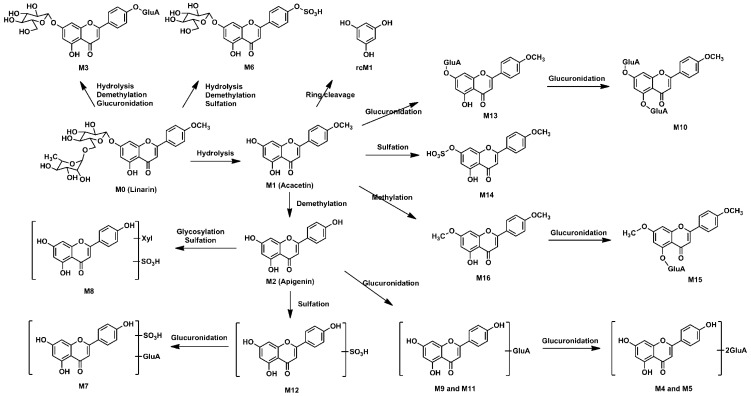
Proposed metabolic pathways of linarin in rats after oral administration. The position of glucuronidation and sulfation metabolism was speculated based on the reactivity of the free hydroxyl groups.

The intestinal flora and the in vivo metabolic pathways of linarin are summarized in Figure 4 and Figure 5, respectively. Linarin was able to be hydrolyzed into acacetin when incubated with rat intestinal flora. Subsequently, demethylation metabolism occurred and apigenin was generated. Apigenin underwent ring fission metabolism and the final degradation metabolites of linarin were 4-hydroxybenzoic acid, 4-hydroxy benzaldehyde and phloroglucinol. Interestingly, of the three ring cleavage metabolites, only phloroglucinol was detectable in rat biosamples. This may be caused by the extremely low responses of these ring cleavage metabolites under the current experiment conditions. In our preliminary study, a mixed stock solution containing 100 μg/mL of 4-hydroxybenzoic acid, 4-hydroxy benzaldehyde and phloroglucinol were prepared in methanol. By successive dilution of the mixed stock solution with methanol, a series of standard working solutions were obtained. For phloroglucinol, a stable MS signal was observed when the concentrations were higher than 1.0 μg/mL. For 4-hydroxybenzoic acid and 4-hydroxy benzaldehyde, stable MS signals were only obtained when their concentrations were higher than 10 μg/mL. These results indicated that 4-hydroxybenzoic acid and 4-hydroxy benzaldehyde might be undetectable in rat biosamples when their concentrations were lower than 10 μg/mL under the current experimental conditions. Thus, a more specific and sensitive analytical method is needed for the determination of rcMs in rat biosamples.

A total of 17 metabolites were identified in rat biosamples. Hydrolysis, demethylation, glucuronidation, sulfation, glycosylation, methylation and ring cleavage were the major metabolic pathways of linarin in rats. As far as we know, this study reports ring cleavage, glycosylation and methylation metabolic pathways of linarin for the first time. For flavone, ring cleavage metabolism usually occurs in intestinal flora and low-molecular-phenolic acids are the major metabolites. In this study, ring cleavage metabolism of linarin was observed and three rcMs were detected. For the methylation metabolism, it is assumed that flavone can be methylated by catechol-*O*-methyltransferase since they contain a catechol moiety [17]. Additionally, apigenin-xyloside was identified as one of the metabolites. Similar glycosylation metabolic pathways have also been reported in other flavonoids, such as icaritin [18]. It is believed that when catalyzed by glycosyltransferases, sugar residue can be transferred to nucleophilic glycosyl acceptor molecules, such as flavonoids, and that the sugar residues are usually derived from activated donors like sugar-nucleotide derivatives (UDP-Glc, UDP-Gal, UDP-GlcNAc, UDP-GalNAc, UDP-Xyl and UDP-GlcA) [19].

Interestingly, our study did not detect the acetylated, hydroxylated and hydrogenated products of linarin that were detected in Tao’s study [10]. This may be because of the species difference. In Tao’s study, intestinal bacteria were derived from human feces, while in this study rat feces were used. Besides the species difference, it is a general belief that no two individuals have the exact same strains of bacteria in their intestinal track [20]. Also, the notable degree of inter-individual variation in bacterial metabolism of flavonoids has been widely reported. Another possible reason that the hydrogenated product of linarin was not detected is that the hydrogenated product of linarin may be a transient intermediate. Quercetin and luteolin, the metabolic pathways of which are well-documented and comparable, are first reduced to hydrogenated products and then undergo ring fission metabolism when incubated with intestinal bacteria. The hydrogenated products of quercetin and luteolin are similarly barely detected since they are transient intermediates [13].

Additionally, it is worth mentioning that, among the 17 metabolites identified, only acacetin, apigenin and the three ring cleavage metabolites were confirmed by comparison with reference standards. The identification of the other metabolites is tentative and further confirmation by comparison with standards is still needed.

## 3. Materials and Methods 

### 3.1. Chemicals and Reagents

Linarin, acacetin and apigenin (purity > 98%) were obtained from Shanghai Winherb Medical Technology Co. Ltd (Shanghai, China). 4-Hydroxybenzoic acid, 4-hydroxy benzaldehyde, phloroglucinol (purity > 98%) and LC/MS-grade formic acid were purchased from Shanghai Macklin Biochemical Co. Ltd. (Shanghai, China). LC/MS-grade acetonitrile was purchased from Fisher Scientific (Pittsburgh, PA, USA). Ultra-pure water was purified using a Milli-Q system (Merk Millipore, Billerica, MA, USA). Other chemicals were of analytical grade.

### 3.2. Preparation of Rat Intestinal Flora

Anaerobic culture medium was prepared as follows [21]: K_2_HPO_4_ (37.5 mL, 0.78%), solution A (37.5 mL, 0.47% KH_2_PO_4_, 1.18% NaCl, 1.2% (NH_4_)_2_SO_4_, 0.12% CaCl_2_, and 0.25% MgSO_4_•H_2_O), Na_2_CO_3_ (25 mL, 8%), l-cysteine (0.5 g), l-ascorbic acid (2 mL, 25%), eurythrol (1 g), and tryptone (1 g) were mixed together and diluted with distilled water to 1 L. The solution was then adjusted to pH 7.5–8.0 with 2 M HCl. Fresh feces collected from Sprague-Dawley rats were immediately homogenized in 0.9% (*w*/*v*) NaCl aqueous solution at a ratio of 1 g to 4 mL. The homogenate was centrifuged (4 °C, Cence L530R, Hunan, China) at 1890 g (equal to 2000 rpm) for 10 min and the supernatant (10 mL) was added to the anaerobic culture medium (90 mL) to obtain an intestinal flora cultural solution.

### 3.3. Metabolism of Linarin in Rat Intestinal Flora

The intestinal flora cultural solution was preincubated for 12 h at 37 °C. Linarin (10 μL, 90 mg/mL, dissolved in dimethyl sulfoxide (DMSO)) was then added to the intestinal flora cultural solution (10 mL). After incubation for 6 and 12 h at 37 °C, 1 mL of the cultural solution was transferred into a clean tube and 5 mL of methanol was added to stop the reaction. The mixture was then vortexed and centrifuged for 10 min. The supernatant was evaporated to dryness under a gentle stream of nitrogen and the residue was reconstituted in 500 μL 50% methanol aqueous solution. A 2 μL aliquot was analyzed by the UPLC/Q-TOF-MS/MS method described below.

### 3.4. Animals and Drug Administration

The animal study was approved by the Animal Ethics Committee of Tianjin University of Traditional Chinese Medicine and carried out according to the Guide for the Care and Use of Laboratory Animals (National Institutes of Health). The approval No. is TJAB-TJU20160031. Male Sprague-Dawley rats weighting 200–220 g were obtained from the Beijing Military Medical Science Academy of the PLA (Beijing, China). Rats were kept in metabolic cages and were fasted with free access to water for a period of 12 h prior to the experiment. The solutions of linarin were prepared in 0.5% CMC-Na and orally administered to rats at a dose of 50 mg/kg once a day for three consecutive days. 

### 3.5. Sample Collection

Following oral administration of linarin on the third day, urine samples were collected during 0–12 h and 12–24 h afterwards, and feces were collected for 0–24 h. For plasma collection, blood was collected from the orbital vein at 0.25, 0.5, 0.75, 1, 1.5, 2, 4, 6, 8 and 12 h after the last drug treatment. Blood samples were then centrifuged (Eppendorf AG 22331, Hamburg, Germany) at 6857× *g* (equal to 8000 rpm) for 10 min (room temperature) to obtain the plasma. For bile collection, the rats were anesthetized by intra-peritoneal injection of 20% ethyl carbamate at the dose of 1.0 g/kg after the last oral administration. An abdominal incision was then made and the common bile duct cannulated with polyethylene tubing to collect bile samples for 0–24 h. All samples were stored at −20 °C until analysis (0–1 month).

### 3.6. Sample Preparation

For urine samples, C18 reversed-phase columns (ODS columns, 500 mg) were prepared and used. An aliquot of 3 mL of mixed urine sample was loaded onto ODS columns preconditioned with 10 mL methanol, and followed by 10 mL water. The cartridges were then eluted with 3 mL of water and 3 mL of methanol successively. The methanol eluate was collected and evaporated to dryness under nitrogen gas at room temperature. The residue was dissolved in 1 mL of a methanol-water mixture (50:50, *v*/*v*). An aliquot of 3 mL of mixed bile samples was treated by the same procedure as the urine samples.

For feces samples, a 1 g dried and powdered feces sample was immersed in 10 mL of methanol and ultrasonically extracted for 30 min. After centrifugation (Eppendorf AG 22331, Hamburg, Germany) at 6857× *g* (equal to 8000 rpm) for 10 min (room temperature), 1 mL of the supernatant was transferred into a clean tube and dried under nitrogen gas at room temperature. The residue was dissolved in 1 mL of a methanol-water mixture (50:50, *v*/*v*).

For plasma samples, an aliquot of 1 mL of mixed plasma sample was vortexed with 5 mL of methanol for 2 min to precipitate proteins. After centrifugation (Eppendorf AG 22331, Hamburg, Germany) at 12,000× *g* (equal to 14,000 rpm) for 10 min (room temperature), the supernatant was separated and evaporated to dryness under nitrogen gas at room temperature. The residue was dissolved in 1 mL of methanol-water mixture (50:50, *v*/*v*).

Before UPLC/Q-TOF-MS/MS analysis, all samples were centrifuged (Eppendorf AG 22331, Hamburg, Germany) at 12,000× *g* (equal to 14,000 rpm) for 10 min (room temperature).

### 3.7. UPLC Conditions and Q-TOF-MS^E^ Analysis

Chromatographic analysis was performed on an ACQUITY^TM^ UPLC I-Class system equipped with a binary solvent system and an autosampler. Samples were separated on an ACQUITY UPLC BEH Shield RP18 column (2.1 × 50 mm, 1.7 μm) eluted with a mixture of 0.1% formic acid aqueous solution (A) and acetonitrile (B). The gradient program was as follows: an initial hold time of 5 min at 5% B, 5–15% B from 5 to 12 min, 15–45% B from 12 to 16 min, 45–75% B from 16 to 18 min, 75–95% B from 18 to 23 min, 95% B from 23 to 25 min, and 95–5% B from 25.1 to 29 min. The flow rate was 0.4 mL/min and the column temperature was set at 40 °C.

The mass spectrometry detection was performed on a Waters ACQUITY SYNAPTTM G2 high-definition mass spectrometry system (Waters, Milford, MA, USA) equipped with an electrospray ionization (ESI) source monitored in positive and negative ion mode. The mass conditions were set as follows: capillary voltage, 2.5 and 2.0 kV in positive and negative ion mode respectively; sampling cone voltage, 30 and 40 V in positive and negative ion mode respectively; source temperature, 100 °C; desolvation temperature, 400 °C; cone gas flow, 50 L/h; desolvation gas flow, 800 L/h. In MS^E^ (elevated-energy setting) mode, the trap collision energy of the low-energy function was set at 0 eV and the ramp trap collision energy of the high-energy function was set at 10–50 eV. In MS^2^ mode, the collision energy was set at 25 eV. For accurate mass acquisition, the mass was corrected using leucine-encephalin via a LockSpray^TM^ interface at a flow rate of 5 μL/min, and monitoring a reference ion for positive ion mode ([M + H]^+^ = 556.2771) and negative ion mode ([M − H]^–^ = 554.2615) to ensure accuracy during MS analysis. All data were acquired and processed with Metabolynx XS software under the operating interface of Masslynx version 4.1 (Waters). Parameter settings for Metabolynx XS software were as follows: analysis time, 0–25 min; mass defect filter, ±40 mDa; mass window, 0.1 Da; maximum tolerance of mass error, 5 ppm; degree of unsaturation, 6 to 15; and the spectrum was above the relative intensity of 2%. The prediction rules of elemental composition were defined as follows: atom numbers of carbon (0–40), hydrogen (0–50), oxygen (0–25), nitrogen (0–5) and sulfur (0–5).

## 4. Conclusions

In this study, an UPLC/Q-TOF-MS/MS method was used for the characterization of linarin metabolites in rat intestinal flora and biosamples. A total of 16 metabolites with an intact flavone skeleton and three ring cleavage metabolites were detected. Linarin was able to be metabolized into low-molecular-weight phenolic acids in intestinal flora and these metabolites were able to be absorbed into blood circulation. Hydrolysis, demethylation, glucuronidation, sulfation, glycosylation, methylation and ring cleavage were the major metabolic pathways for linarin. This is the first time that ring cleavage, methylation and glycosylation metabolism of linarin has been reported and these results provide scientific support for the further development of linarin as a drug candidate.

## Figures and Tables

**Table 1:** Ultra-performance liquid chromatography coupled with quadrupole time-of-flight mass spectrometry (UPLC/Q-TOF-MS/MS) retention times and fragment ions of the metabolites of linarin in rat biosamples.

No.	RT (min)	Molecular ions [M+H]^+^		ppm	Formula	MS/MS Fragment	Metabolite Description	Location
		Mea.	Cal.					
Metabolites derived from linarin								
M0^a^	14.16	593.1869	593.1870	−0.2	C_28_H_32_O_14_	447.1293, 285.0763	linarin (parent)	P, F, B, I
M1 ^a^	16.01	285.0771	285.0763	2.8	C_16_H_12_O_5_	270.0529, 242.0579, 153.0183, 133.0649	hydrolysis (acacetin)	P, U, F, B, I
M2 ^a^	14.50	271.0606	271.0606	0.0	C_15_H_10_O_5_	242.0572, 153.0180, 119.0491	hydrolysis and demethylation (apigenin)	U, F, I
M3	7.03	609.1445	609.1456	−1.8	C_27_H_28_O_16_	271.0604, 447.0918	hydrolysis, demethylation and glucuronidation	U
M6	9.67	513.0697	513.0703	−1.2	C_21_H_20_O_13_S	271.0606, 351.0166	hydrolysis, demethylation and sulfation	U
Metabolites derived from acacetin (the aglycone of linarin)								
M4	7.18	623.1230	623.1248	−2.9	C_27_H_26_O_17_	271.0604, 447.0928	demethylation and 2×glucuronidation	U, B
M5	8.61	623.1332	623.1248	−2.6	C_27_H_26_O_17_	271.0603, 447.0919	demethylation and 2×glucuronidation	U, B
M7	10.98	527.0495	527.0496	−0.2	C_21_H_18_O_14_S	271.0606, 351.0175, 447.0938	demethylation, glucuronidation and sulfation	U, B
M8	11.05	483.0601	483.0597	0.8	C_20_H_18_O_12_S	271.0606, 351.0171	demethylation, glycosylation and sulfation	U, B
M9	11.11	447.0923	447.0927	−0.9	C_21_H_18_O_11_	271.0605	demethylation and glucuronidation	U, B
M10	12.36	637.1399	637.1405	−0.9	C_28_H_28_O_17_	285.0768, 461.1090	2×glucuronidation	U, B
M11	12.57	447.0919	447.0927	−1.8	C_21_H_18_O_11_	271.0605, 153.0184	demethylation and glucuronidation	U, B
M12	13.70	351.0173	351.0175	−0.6	C_15_H_10_O_8_S	271.0604	demethylation and sulfation	U, F
M13	14.24	461.1083	461.1084	−0.2	C_22_H_20_O_11_	285.0765	glucuronidation	P, U, B
M14	15.10	365.0317	365.0331	−3.8	C_16_H_12_O_8_S	285.0777, 270.0531, 242.0652, 133.0989	sulfation	P, U, F, B
M15	15.21	475.1241	475.1240	0.2	C_23_H_22_O_11_	285.0754	methylation and glucuronidation	U
M16	17.38	299.0924	299.0919	1.7	C_17_H_14_O_5_	284.2952	methylation	F
Ring cleavage metabolites*								
rcM1 ^a,^*	0.57	125.0245	125.0239	4.8	C_6_H_6_O_3_	−	ring cleavage	U, B, I
rcM2 ^a,^*	1.76	137.0237	137.0239	−1.5	C_7_H_6_O_3_	93.0333	ring cleavage	I
rcM3 ^a,^*	2.74	121.0285	121.0290	−4.1	C_7_H_6_O_2_	−	ring cleavage	I

Footnote: P: Plasma; U: Urine; F: Feces; B: Bile; I: Intestinal flora.^a^ Compared with reference standards. * Molecular ions and mass (MS/MS) fragments for these metabolites were obtained in negative ion mode.

## Supplementary Materials

The following are available online at http://www.mdpi.com/1420-3049/23/9/2140/s1, Figure S1: Extracted ion chromatograms (EICs) of linarin metabolites in blank rat biosamples.
