# Supplementary material for: Characterization of the In Vivo and In Vitro Metabolites of Linarin in Rat Biosamples and Intestinal Flora Using Ultra-High Performance Liquid Chromatography Coupled with Quadrupole Time-of-Flight Tandem Mass Spectrometry
Source: Molecules. 2018 Aug 25;23(9):2140. doi: 10.3390/molecules23092140 (PMC6225362; doi:10.3390/molecules23092140)
Supplement: Supplementary file 1 [file molecules-23-02140-s001.pdf]

Supporting Information for

Characterization of the *in vivo* and *in vitro* metabolites of linarin in rat biosamples and intestinal flora using ultra-high performance liquid chromatography coupled with quadrupole time-of-flight tandem mass spectrometry

Xinchi Feng[1,†], Yang Li[1,2,†], Chenxi Guang[1,2], Miao Qiao[1,2], Tong Wang[1,2], Liwei Chai[1,2] and Feng Qiu[1,2,*]

[1] School of Chinese Materia Medica, Tianjin University of Traditional Chinese Medicine, Tianjin 300193, China

[2] Tianjin State Key Laboratory of Modern Chinese Medicine, Tianjin University of Traditional Chinese Medicine, Tianjin 300193, China

[*] Correspondence: fengqiu20070118@163.com; Tel.: +86-22-5959-6223

[†] These authors contributed equally to this work.

Supplemental Figures

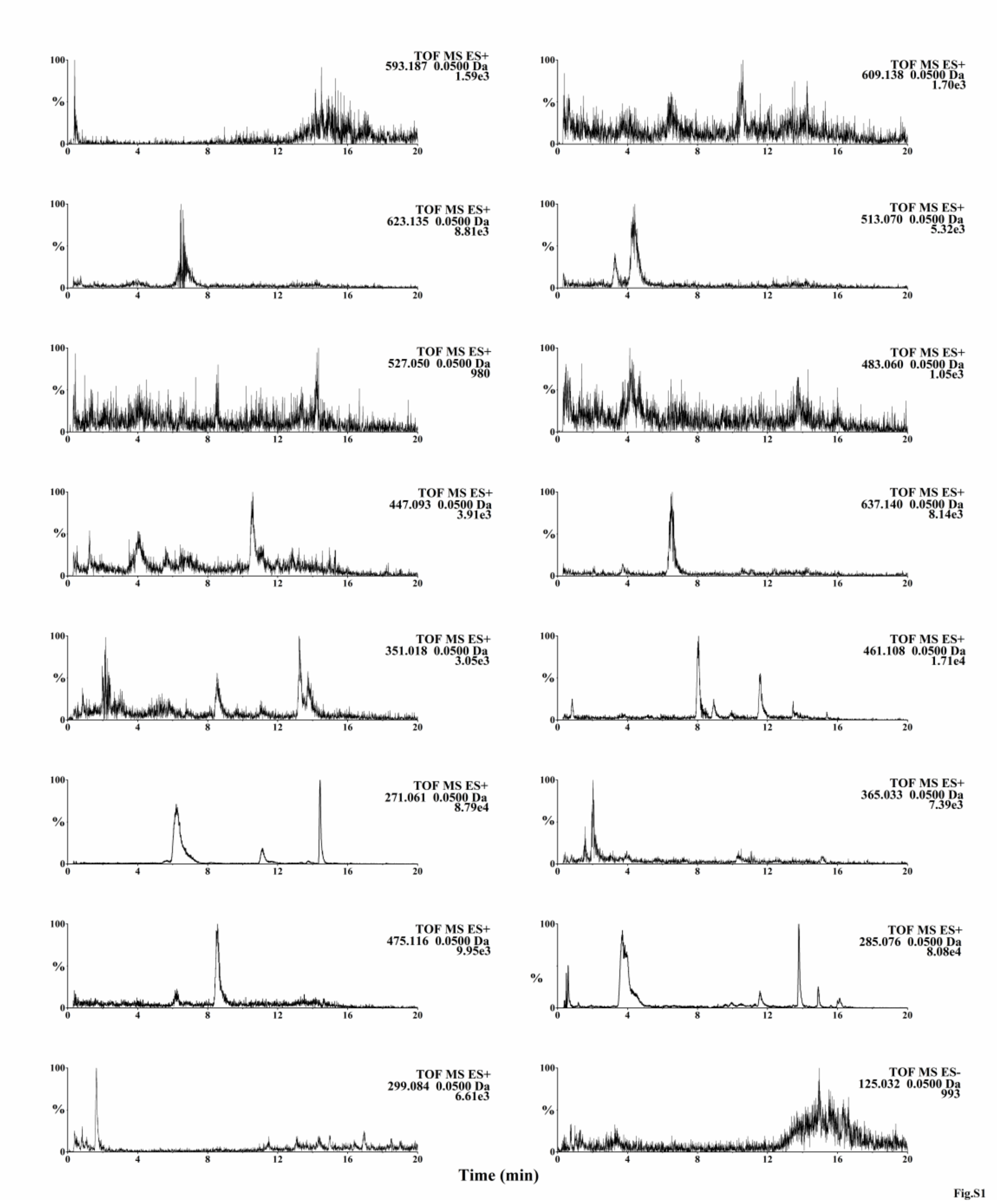

Figure S1 Extracted ion chromatograms (EICs) of linarin metabolites in blank rat biosamples.
